# An Interferometric Multi-Sensor Absolute Distance Measurement System for Use in Harsh Environments

**DOI:** 10.3390/s25175487

**Published:** 2025-09-03

**Authors:** Mateusz Sosin, Juan David Gonzalez Cobas, Mohammed Isa, Richard Leach, Maciej Lipiński, Vivien Rude, Jarosław Rutkowski, Leonard Watrelot

**Affiliations:** 1 CERN European Organization for Nuclear Research, 1211 Geneva, Switzerland; juan.david.gonzalez.cobas@cern.ch (J.D.G.C.); leonard.watrelot@cern.ch (L.W.); 2 Manufacturing Metrology Team, Faculty of Engineering, University of Nottingham, Nottingham NG7 2RD, UK; mohammed.isa@nottingham.ac.uk (M.I.);; 3 Leica Geosystems AG, 9435 Heerbrugg, Switzerland

**Keywords:** frequency sweeping interferometry, frequency scanning interferometry, accelerator alignment, robust sensor, distance measurement

## Abstract

Fourier transform-based frequency sweeping interferometry (FT-FSI) is an interferometric technique that enables absolute distance measurement by detecting the beat frequencies from the interference of reflected signals. This method allows robust, simultaneous distance measurements to multiple targets and is largely immune to variations in the reflected optical signal intensity. As a result, FT-FSI maintains accuracy even when measuring reflectors with low reflectance. FT-FSI has recently been integrated into the full remote alignment system (FRAS) developed for the High-Luminosity Large Hadron Collider (HL-LHC) project at CERN. Designed to operate in harsh environments with electromagnetic interference, ionizing radiation and cryogenic temperatures, FRAS employs FT-FSI for the precise monitoring of the alignment of accelerator components. The system includes specialized interferometers and a range of sensors, including inclinometers, distance sensors, and leveling sensors. This paper presents a comprehensive review of the challenges associated with remote measurement and monitoring systems in harsh environments such as those of particle accelerators. It details the development and validation of the FT-FSI-based measurement system, emphasizing its critical role in enabling micrometric alignment accuracy. The developments and results presented in this work can be readily translated to other demanding metrology applications in harsh environments.

## 1. Introduction

Particle accelerators were first developed in the early 20th century as physicists sought to investigate the atomic and subatomic properties of matter [1,2]. Since then, accelerator technology has continuously evolved, presenting complex challenges in system design and integration to engineers. To achieve higher beam energies and improved performance, modern accelerators incorporate advanced technologies, such as high-magnetic-field superconducting magnets and superconducting radio frequency acceleration cavities that require cryogenic cooling [3]. Maintaining reliable and robust system functionality when integrating these components remains a significant engineering challenge.

In addition to the technical challenges linked to such technologies, accelerators also expose their components to high levels of ionizing radiation. This radiation, generated by interactions between charged particles and surrounding materials, can lead to material degradation, accelerated corrosion, lubricant breakdown, and polymer deterioration. Furthermore, electronic control systems are particularly susceptible to radiation effects and damage [4,5,6,7,8,9]. These extreme conditions make the design of accelerator instrumentation very demanding.

The integration of accelerator system components is typically further complicated by stringent alignment requirements essential to guarantee proper machine operation. Misalignments between neighboring magnetic elements can introduce local perturbations in the motion of the accelerated particles that can degrade accelerator performance and increase radiation levels through the loss of particles from the accelerator. Typical alignment accuracy requirements range from sub-millimeter accuracy over distances of several hundred meters to millimeter accuracy over distances of tens of km [3,10]. Future accelerator projects, such as the Compact Linear Collider (CLIC) [11] and the Future Circular electron-positron Collider (FCC-ee) [12], demand even higher alignment accuracy down to the micrometre level at the collision points.

Achieving the required alignment tolerances necessitates specialized techniques and high-precision instrumentation. In environments where human access is feasible, typical surveying instruments such as laser trackers are commonly employed [10]. Challenges arise in regions where radiation levels, cryogenic systems, or vacuum constraints restrict personnel access, or where alignment tolerances are too stringent to be maintained with conventional survey equipment. In such cases the only viable solution is to equip accelerator components with position measurement sensors and remote adjustment mechanisms that can operate in such harsh environmental conditions, minimizing the need for human intervention. An example of such a system is the Large Hadron Collider (LHC) inner triplet monitoring system for magnets that focus beams into a tight spot at the center of each of the four LHC experiments [13]. This has been operational since 2009. The system was designed to monitor the stability of the final focus quadrupole magnets with the possibility to remotely re-align them when necessary, with micrometric precision. It can function in radiation levels exceeding 16 kGy/year and strong magnetic fields. To do so, it uses radiation- and magnetic-resistant materials, mainly metals and ceramics. It relies on capacitive sensors for its wire positioning system (WPS) and hydrostatic leveling system (HLS) due to their simple construction and the ability to read them out through long cables, which allows their acquisition electronics to be placed in low-radiation areas.

Since 2015, CERN surveyors and engineers have been developing a new method for the full remote alignment of key components of the High Luminosity Large Hadron Collider (HL-LHC) project [14,15]. The full remote alignment system (FRAS) [16] instrumentation was designed to operate in an even more challenging environment than the LHC, providing micrometric measurements under conditions that include high vacuum, cryogenics, proximity to strong magnetic fields, and exposure to radiation levels reaching up to a 5 MGy total ionizing dose (TID).

**Figure 1:**
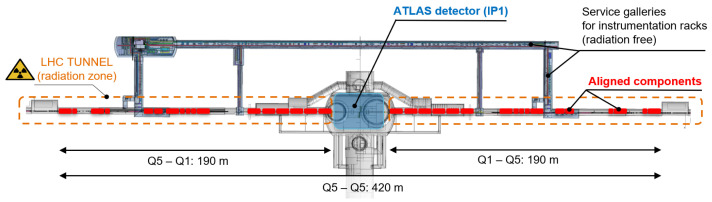
FRAS system for the alignment of the zone around interaction point 1 (ATLAS experiment) of the HL-LHC.

Figure 1 presents a simplified layout of the FRAS system on both sides of interaction point 1 (IP1) of the HL-LHC, where the ATLAS experiment is situated. A similar installation is foreseen on both sides of the CMS experiment at interaction point 5 (IP5). A total of 34 components per IP will be continuously monitored and can be remotely aligned through electronics located in the radiation-free service galleries parallel to the LHC tunnel. Ensuring optimal alignment using the FRAS has reduced the need for magnetic correction elements in this region, lowering the overall cost of the project. It will also limit the radiation exposure for surveyors intervening in these regions.

The primary objective of the FRAS is to align all 34 components on both sides of the IP (from Q5 left to Q5 right) over a 420 m-long zone (see Figure 1), ensuring that their axes remain within an elliptical standard deviation tolerance zone (±1σ) of 0.34 mm in the vertical direction and 0.66 mm in the radial (horizontal) direction. Additionally, all the 17 FRAS components on one side of the experimental area must be positioned within a cylindrical standard deviation tolerance zone (±1σ) with a diameter of 0.2 mm.

**Figure 2:**
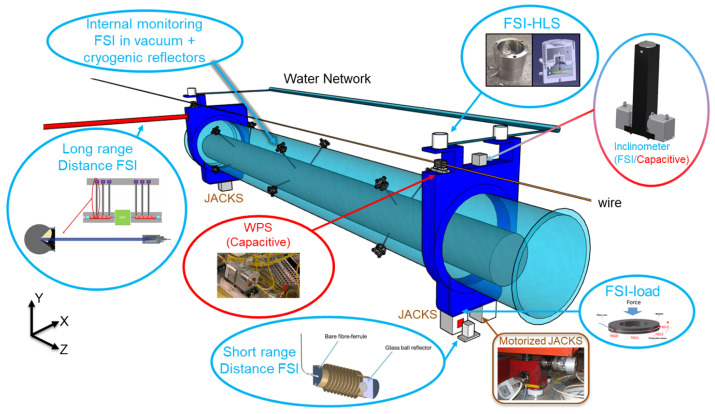
FRAS sensors installed on a magnet.

The remote determination of component positions is achieved using a redundant configuration of sensors (see Figure 2):

- Wire positioning sensors (WPS): X–Y distance sensors used to determine the vertical and radial offsets of components relative to a stretched wire.
- Hydrostatic leveling sensors (HLS): These measure the vertical offset to a water surface, serving as a leveling reference to determine component positions in the vertical direction.
- Inclinometers: These measure the roll angle of the aligned components.
- Distance monitoring sensors: These can be short-range (<200 mm), monitoring the longitudinal stability of component positions, or long-range (<16 m), measuring the distance to the straight reference WPS line of the side galleries.
- Internal monitoring optical vacuum heads (feedthroughs) and cryogenic reflectors: These measure the distance between special vacuum heads installed on the cryostat (at ambient temperature) and the reflectors that are at cryogenic temperatures and attached to the cold mass of the magnet or radio frequency cavities (further details on the internal monitoring application can be found in Section 3.3).

To meet the aforementioned alignment requirements, the measurement uncertainties for all distance sensors must be less than 5 μm, except for the long-range sensor, which has a required uncertainty of 20 μm. The required uncertainty for the angular sensor is 15 μrad. These uncertainty values apply to all sensor measurements at a confidence level of 68% (k = 1).

The primary challenge in designing and deploying the FRAS sensors was to ensure that they could deal with the harsh accelerator environment in which they must operate. The position-monitoring sensors must withstand a total ionizing dose (TID) of 1 MGy [17], with even higher radiation conditions expected for the cryogenic retro-reflectors on the cold masses, where radiation levels could reach 5 MGy. The sensors must maintain the required measurement accuracy for the 15-year operational lifetime of the HL-LHC, and ideally be maintenance-free. All components of the internal monitoring system must function under vacuum conditions and cryogenic temperatures.

Achieving micrometric accuracy while optimizing system costs poses another significant challenge, primarily due to the large number of required sensors. The system incorporates 276 WPS sensors, 148 HLS sensors, 36 inclinometers, 66 longitudinal measurement sensors, and 320 non-contact optical measurements within the cryostats. This results in a total of 904 sensors to be deployed.

After intensive testing between 2016 and 2019, Fourier transform-based frequency scanning interferometry (FT-FSI) was chosen for all sensors except the WPS [18,19]. WPS sensors use capacitive technology, which could not be replaced with an optical alternative at that time.

This paper introduces the FT-FSI technique, outlining the fundamental principles of interferometer operation and its advantages for robust, multi-sensor absolute-distance measurement systems. Previous works from our group have covered the FT-FSI principle, interferometer configurations, and early FRAS sensor applications, quantifying their measurement performance (resolution, accuracy, and stability) and introducing their initial implementation [18,19,20,21,22]. Building on that foundation, this article presents, for the first time, finalized, robust sensor designs and a validated multi-sensor framework engineered for reliable, long-term operation in harsh accelerator environments.

It details the sensor concepts and final design, highlighting the challenges encountered, the solutions developed, and the achieved outcomes. In addition, it summarizes the verification results and recent upgrades prior to FRAS deployment during the LHC’s third long shutdown (LS3, 2026–2029). To guide readers, Section 1 provides a new integrative review of the challenges of accelerator alignment in harsh environments and frames the HL-LHC FRAS context, Section 2 offers a condensed review of FT-FSI fundamentals, and Section 3 and Section 4 report the novel engineering solutions and validation data specific to sensor operation.

## 2. Materials and Methods: FT-FSI Measurement System

**Figure 3:**
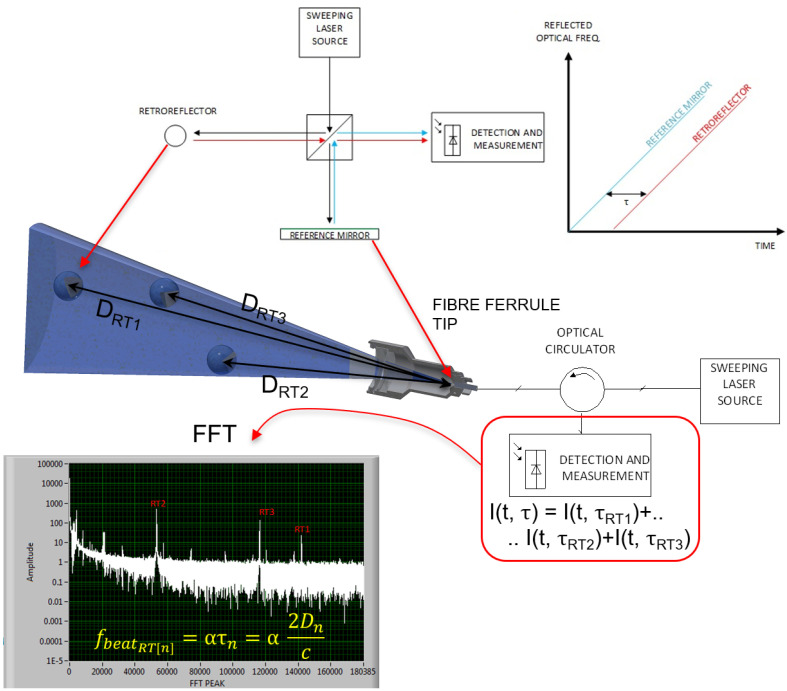
FT-FSI operation principle. Adapted from [18].

The principle of operation of the FT-FSI measurement setup is similar to the Michelson interferometer, where a constant frequency laser is replaced by a swept laser source (see Figure 3). The swept signal from the laser source (via a reference mirror) is combined with the optical signal reflected back from a retroreflector at a photodetector. The “time of flight delay” of the two signals leads to a beat frequency in the interference signal [18,23,24], according to Equation (1):

(1) $$I\left(t,\tau \right)=Acos[2\pi \left(\alpha \tau t+f_{0}\tau \right)],$$

where *A* is the magnitude of the signal; τ is the time delay between the signal from the reflecting target ($RT_{n}$) and the reference mirror at the photodetector (see Figure 3); α is the sweep rate of the laser ($\alpha \;=\frac{d\nu }{dt}$ is the laser frequency change in time); $f_{0}$ is the optical frequency of the laser at time $t_{0}$.

To simplify the interferometer design, a configuration using a circulator and a fiber ferrule tip is commonly employed (see Figure 3). In this setup, the fiber ferrule tip functions as both a beam splitter and a reference mirror, reflecting approximately 4% of the incident light [25] to form the reference arm. The remaining 96% is directed towards the reflective target, often using collimating optics.

When retroreflectors are placed in the beam path, a portion of the reflected light is incident on the fiber and can be directed to a photodiode using a circulator. Here, it combines with the reference beam to generate an interference pattern. The photodetector’s electrical response to this signal can be described by Equation (2):

(2) $$\begin{array}{cc} I(t,\tau )=\\ & A_{RT1}\cdot cos\left[2\pi \left(\alpha \tau_{RT1}t+f_{0}\tau_{RT1}\right)\right]+A_{RT2}\cdot cos\left[2\pi \left(\alpha \tau_{RT2}t+f_{0}\tau_{RT2}\right)\right]+\\ & A_{RT3}\cdot cos\left[2\pi \left(\alpha \tau_{RT3}t+f_{0}\tau_{RT3}\right)\right]+A_{n}\cdot cos\left[2\pi \left(\alpha \tau_{RTn}t+f_{0}\tau_{RTn}\right)\right]+\ldots \end{array},$$

where indexes RT1,RT2,RT3…n distinguish the parameters for each retroreflector (cf. Figure 3).

When the laser sweeping speed α is constant, each detected interference signal becomes a constant beat frequency dependent on the distance $D_{n}$ to the targets (Equations (3) and (4)). By applying the fast Fourier transform (FFT), we can easily determine these beat frequencies and convert them to the corresponding distances (cf. Figure 3).

(3) $$f_{beat_{n}}=\alpha \tau_{n}=\alpha \;\frac{2D_{n}}{c},$$

(4) $$D_{n}=c\frac{f_{beat_{n}}}{2\frac{d\nu }{dt}}.$$

The primary advantage of this measurement method is its insensitivity to variations in signal intensity, unlike classic interferometric techniques that rely on phase measurements. In addition, it enables the simultaneous measurement of multiple distances using a single interferometer channel.

Due to imperfections in tunable external cavity diode laser (ECDL) sources that arise from inaccuracies in their opto-mechanical layout [26,27,28], achieving micrometric measurement accuracy with FT-FSI is challenging. These imperfections introduce non-linearities into the sweep parameter α, and consequently in all measured beat frequencies. To compensate for this effect, a linearization process is required [18]. Tracking the sweep frequency is also essential for computing the measured distances [18,20].

**Figure 4:**
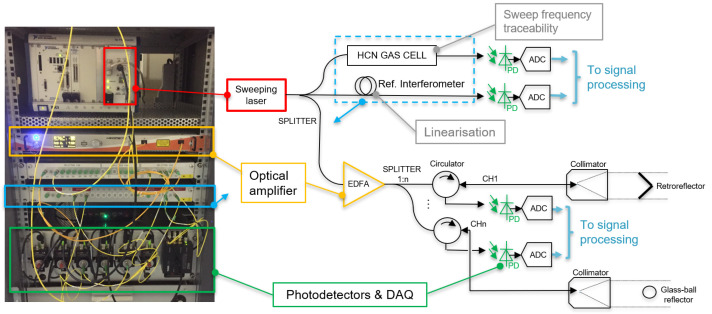
CERN FT-FSI setup: first prototype (2017).

To address these challenges, all the CERN FT-FSI measurement systems (designed and manufactured internally at CERN, Geneva, Switzerland) (see Figure 4) are equipped with the following sub-components:

- A reference interferometer is a constant-length interferometer used for linearizing the optical sweep, mitigating unwanted modulations that affect measurements.
- An absorption gas cell [29] is used to track the “true” frequency of the sweeping laser [30,31].
- An erbium-doped fiber amplifier (EDFA) is used to ensure appropriate amplification of the laser signal before distribution to multiple measurement channels.
- Measurement channel circuitry consists of an optical circulator, measurement optics (collimator or bare ferrule), a photodetector, and a data acquisition system (DAQ), providing measurement data to a processing computer.

The FT-FSI setup offers an additional valuable advantage: it enables the measurement of Bragg grating-based sensors [32]. These sensors operate by reflecting specific wavelengths of light, which shift in response to changes in the grating’s periodic structure. Variations in fiber strain, caused by temperature or other factors, alter the grating period, thereby modifying the reflected or transmitted wavelength. These changes can be precisely detected by optical spectrometers. By utilizing the FT-FSI gas cell to determine the exact frequency of the sweep laser, the interferometer can effectively function as an optical spectrometer.

**Figure 5:**
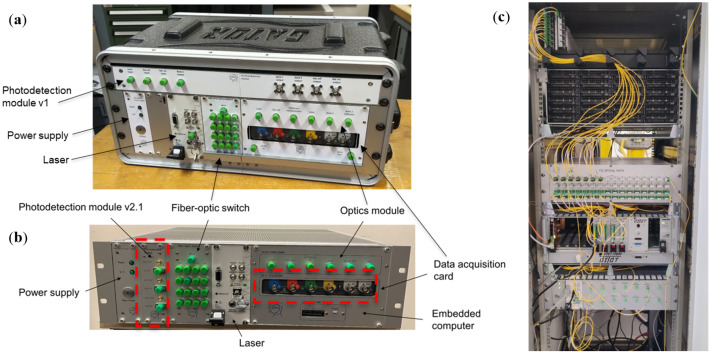
(**a**) Portable FT-FSI unit v.1; (**b**) Portable unit v.2; (**c**) 256-channel interferometer rack prototype.

The CERN FT-FSI setup was developed based on an analysis of prior research [23,33,34]. The core layout, introduced in the first interferometer prototype (depicted in Figure 4), has remained unchanged across all generations of FT-FSI interferometers used at CERN:

- **Portable FT-FSI measurement units:** During the development and testing of the FRAS internal monitoring system (2017–2022), an increasing number of tests in various locations and the demand for additional measurement channels led to the creation of portable FT-FSI units (see Figure 5a,b). These units, built upon the experience from the initial prototype, improved portability, expanded measurement capabilities, and optimized the cost of signal acquisition circuitry.
- **FRAS production interferometers:** The FRAS system requires over 660 optical channels for its interferometric sensors (see Section 1), along with 140 Bragg-based load sensors for supporting jack mechanics, bringing the total number of optical channels to more than 800. To manage this large-scale setup and allow for future expansion, FT-FSI data acquisition is distributed across four independent racks (see Figure 5c). Each rack supports up to 248 measurement channels, collectively providing a capacity of 992 channels. These racks are strategically positioned on the left and right sides of the IP1 and IP5 service galleries of the LHC (cf. Figure 1), ensuring full coverage of the FRAS installation while allowing room for future upgrades.

Further details regarding the interferometer construction approach, testing procedures, and performance analysis can be found in [18,20,21,22]. The results of the FT-FSI measurement unit tests are summarized in Section 4.1.

The adoption of FT-FSI for the FRAS measurement system was driven by five key advantages. First, it enables absolute distance measurements, ensuring a reliable reference for precision alignment. Second, unlike conventional interferometric methods, FT-FSI is highly tolerant to variations in light intensity, maintaining accuracy even in challenging accelerator environments. Third, it eliminates issues related to electromagnetic noise, which previously affected capacitive sensors in CERN’s monitoring systems. Fourth, the use of optical fibers significantly reduces infrastructure costs compared to the cables required for capacitive-sensor-based setups. Finally, FT-FSI requires no sensor recalibration, relying on fundamental optical principles that enhance long-term stability and reduce maintenance requirements.

These advantages make FT-FSI an optimal choice for alignment monitoring in harsh environments, facilitating the implementation of non-contact “internal monitoring” applications and enabling the design of cost-optimized optical sensors, including HLS, inclinometers, and distance sensors.

## 3. Materials and Methods: Sensors

### 3.1. Sensor Material Environmental Compatibility

As introduced in Section 1, FRAS sensor technology must be compatible with radiation, temperature, and vacuum constraints while ensuring simplicity and robustness in operation.

The main challenge in the LHC environment is to ensure resistance to the effects of ionizing radiation, in particular the total ionizing dose (TID), which can reach 5 MGy [4,6].

To ensure resistance to such high TID values, material selection for sensor design focused primarily on radiation hardness. The sensor bodies are manufactured from austenitic stainless steel (304L or 316LN), which is highly resistant to radiation and benefits from a natural protective chromium-oxide layer that prevents oxidation. Aluminium with an anodized layer for oxidation protection was also employed. Austenitic stainless steel and aluminium were also chosen for their low magnetic permeability, ensuring minimal interaction with strong magnetic fields. All thermoplastics and epoxy fillers were selected from materials validated by CERN for radiation environments [35,36].

The optical sub-components used (glass reflectors, mirrors, fiber feedthroughs, fibers, etc.), as well as the optical assemblies, were tested during several irradiation campaigns in 2015, 2018, and 2022 to qualify their performance under radiation. The verification of difficult-to-estimate aging effects was addressed by testing the components at a TID level at least twice as high as that expected during HL-LHC operation. These tests confirmed the performance of the selected materials before their approval as final materials for the FRAS sensors [36,37].

In addition, regarding the darkening of glass in optical components, sensitivity to this effect was mitigated using a near-infrared laser (optical C-band), for which glass attenuation is lower compared to visible wavelengths [38,39,40]. Moreover, specially developed CERN radiation-hardened fiber patch cords were be used to further minimize radiation effects in high-radiation areas [41,42].

The cryogenic compatibility of reflective targets and epoxy resins, as well as their thermo-mechanical properties, was carefully studied before finalizing their design [43]. All reflectors were tested for performance across multiple cool-down cycles, and their mechanical clamping solutions were validated for repeatable positioning throughout these cycles.

Regarding vacuum compatibility, all sealing solutions (gaskets and fiber feedthroughs) use materials already validated at CERN for radiation resistance [4,6,35,36].

### 3.2. Sensor Design Approach: Enhanced Robustness Through Simplicity

Apart from the selection of sensor materials based on the need for compatibility with harsh environments, ensuring that the sensors remain simple and robust in operation is equally essential to maximize their potential to be maintenance-free. This can be achieved on a large scale through simplification and minimising the number of components that could potentially fail.

For FRAS, the adoption of FT-FSI technology enabled a significant simplification of sensor construction, allowing the use of divergent laser beams combined with high-refractive-index glass ball reflectors. This reduced complexity while maintaining precision and reliability.

The use of divergent laser beams increased the laser beam footprint at the reflector, enabling accurate measurements even when the reflector undergoes several centimeters of lateral displacement. As a result, the optical components used to launch the laser beam could be integrated without adjustment mechanisms. Alignment and readjustment needs were therefore eliminated, reducing the overall system complexity.

**Figure 6:**
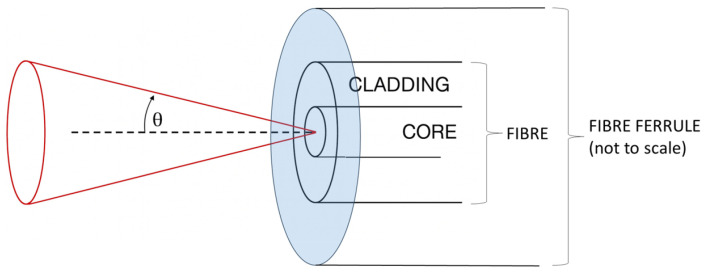
Divergent beam launched from the fiber, as defined by the numerical aperture of the fiber.

The simplest, most robust, and cost-effective solution to launch the divergent beam without the use of lenses is to use a bare single-mode fiber tip, where the polished bare fiber tip becomes the source of a conical Gaussian beam [44]. The numerical aperture (NA) of the fiber then defines the divergence cone of the output laser beam. The beam divergence angle (θ, cf. Figure 6) can be retrieved from Equation (5):

(5) $$NA=n\cdot sin\left(\theta \right)\to \theta =arcsin\left(\frac{NA}{n}\right),$$

where *n* is the index of refraction of the medium through which the beam propagates. For the selected Corning SMF28 fiber, NA = 0.14 [45] (θ=8°); hence, the useful, full divergence angle of the beam is around 16°. The bare fiber ferrule, with its flat, polished surface, also acts as the interferometer mirror/splitter (see Section 2). The use of bare ferrules also allows for easy calibration, as coordinate measurement machines (CMM) can easily measure the ferrule cylinder to determine the fiber tip (semi-transparent mirror), which is located in the center of the ferrule.

**Figure 7:**
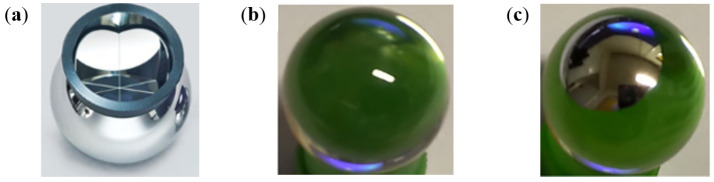
Three different types of reflectors: (**a**) commercial 0.5″ SMR; (**b**) 0.5″ TAFD 55 glass ball; (**c**) 0.5″ TAFD glass ball with a reflective coating.

The second challenge of the FRAS system was the need for permanent monitoring of hundreds of optical reflectors. Initially, the use of commercially available spherically mounted retroreflectors (SMRs) (see Figure 7a) was considered. However, these reflectors are very expensive due to their tight machining tolerances and precise pre-sale characterization. Additionally, FRAS optical reflectors needed to be radiation-hard. To reduce overall system costs and increase reliability, a low-cost reflector was developed, consisting of a high-refractive-index glass bead (*n* = 2) with simple coating. Using high-index glass ensures that incident rays of light are internally refracted and reflected by 180 degrees such that they exit towards the light source, effectively acting like a typical SMR [46]. For the FRAS system, the reflectors are precisely machined TAFD55 glass balls (see Figure 7b,c) and can be equipped with a reflective coating on the backside to enhance the reflected signal intensity. The TAFD55 glass reflectors were subject to intensive radiation testing, demonstrating good performance under the expected radiation conditions.

It is important to note that most of the glass reflectors used in the FRAS are 0.5″ diameter balls. This ensures compatibility with classical 0.5" SMRs used for laser tracker measurements and is crucial during the fiducialization process [47], e.g., when determining the position of a component relative to the reflector’s position. After carrying out laser tracker measurements using SMRs, the same reflector nests (fiducials) are used to install the glass ball reflectors, providing the necessary link between fiducialized geometries and FT-FSI measurements.

The measurement configuration, consisting of a bare ferrule for beam emission (using a 1 mW laser output) and *n* = 2 glass reflectors (with diameters ranging from 4 to 20 mm), was tested across various distances from 0.05 to 0.7 m, which is typical for most FRAS applications. The reflection peaks were consistently sharp and distinguishable from the background noise in the FFT spectrum, indicating good system performance [18]. The distance to be measured can be extended by increasing the laser power, although this can introduce additional safety constraints and suffer from parasitic reflections from surrounding surfaces.

### 3.3. FRAS Internal Monitoring Sensors

**Figure 8:**
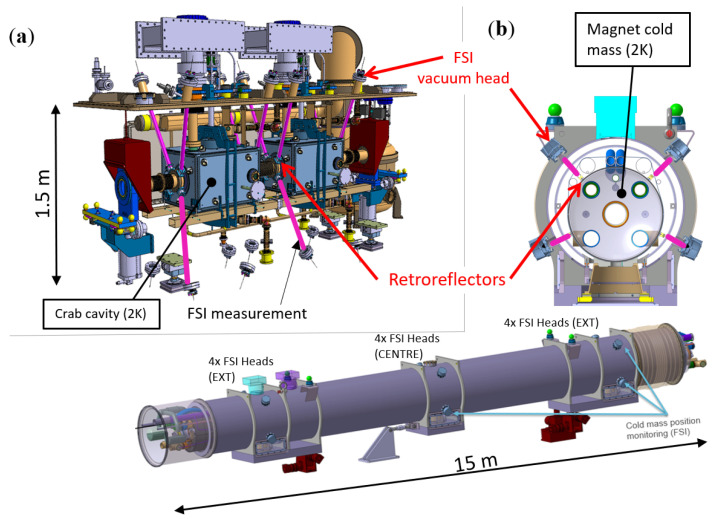
FRAS internal monitoring of crab cavities and magnet cold masses. (**a**) Crab cavity cryostat: this system uses an FSI (frequency scanning interferometry) setup to monitor the internal position of two crab cavities. Each cavity is equipped with 8 FSI heads and 8 retroreflectors. (**b**) Inner triplet magnet application: this employs 12 FSI heads and retroreflectors to monitor various positions along the 15-meter length cold mass of the magnet.

Key FRAS components, such as inner triplet quadrupole magnets (Q1–Q3, used for focusing particle beams to a tight spot within LHC experiments) and the radio-frequency crab cavities (used to tilt particle beams in a crabbing fashion to provide an optimal overlap of the two colliding beams at the interaction point), were be equipped with an internal monitoring system [48] (see Figure 8). This tracks the position of the internal cold sub-components with respect to their external support structures.

As these components operate below 2 K, their position must be measured from outside the external vacuum vessel (cryostat) at ambient temperature using non-contact methods to avoid thermal losses. The monitoring system uses multiple frequency scanning interferometry (FSI) distance measurements. For inner triplet magnets, four optical measurements at three longitudinal positions are performed on target reflectors mounted on the cold mass via dedicated feedthroughs (FSI heads (see Figure 8b)). Each CRAB cavity is monitored using eight distance measurements, four at the entry and four at the exit of the cavities (see Figure 8a) [48].

With the known positions of the FSI heads and the reflectors, and the measured distances between them, we can precisely determine the position, orientation, and thermal contraction of the internal components within the cryostat’s external reference frame.

**Figure 9:**
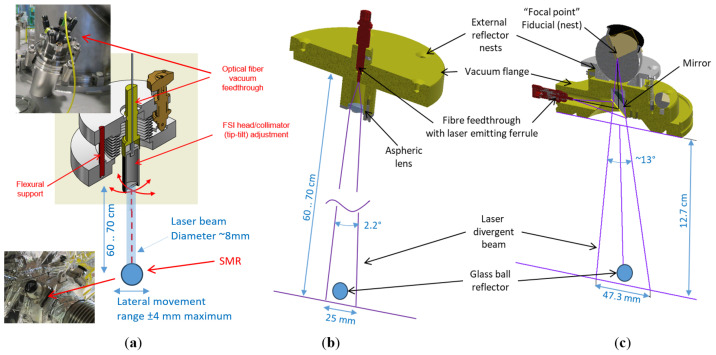
FT-FSI measurement head for (**a**) the crab cavity (the first prototype with a tip–tilt mechanism); (**b**) the crab cavity (divergent beam: no moving parts); (**c**) the inner triplet magnet.

Before the FT-FSI system became available at CERN, a classical FSI system (Etalon Multiline [49]) was used to track distances to a single reflector using a collimated laser beam. This required the use of a specifically designed FSI optical vacuum head with tip–tilt adjustment (cf. Figure 9a) to ensure precise alignment with the SMR retroreflector, compensating for lateral thermal motions within the optical limit range of up to ±4 mm.

Thanks to the low sensitivity of FT-FSI to variations in reflected optical signal intensity and its use of divergent beams, it was possible to design much simpler and robust FT-FSI measurement heads (cf. Figure 9b,c).

For the crab cavity application (see Figure 9a,b), where the retroreflector is located 60 cm to 70 cm from the vacuum port, the beam launched from the fiber ferrule was collimated to a divergence angle of 2.2° using a single aspheric lens (see Figure 9b). This configuration achieved a beam footprint of 25 mm at the reflector, which was sufficient enough to accommodate lateral shifts induced by thermal contraction (approximately ±2 mm) and mechanical assembly errors, thereby eliminating the need for any adjustment mechanism. The entire optical system was integrated into a single metallic head (vacuum flange), and multiple external nests were included to define the ferrule’s “focal point” position from outside the vacuum flange.

For the inner triplet application (see Figure 8b), a key challenge was to manage the cold mass’s significant contraction during cool-down. Since the cold mass is rigidly attached to the vacuum vessel at the center, it is the outer FSI heads that must manage lateral reflector displacements of up to 12 mm. To accommodate this, the inner triplet measurement head (Figure 9c) uses a 12°–16° divergent beam launched from the fiber ferrule, offering a 47 mm laser footprint at these outer reflectors, which is sufficient enough to cover the full range of thermal motion without requiring additional adjustment mechanisms. As with the crab cavity design, the optical system is housed in a compact, cost-optimized metallic body. A mirror was used to reduce the FSI head’s vertical profile and align the external SMR centre with the ferrule’s virtual focal point, enabling interferometer origin determination from outside the vacuum flange. This single-SMR setup was later enhanced with multiple external nests, improving redundancy and eliminating the need for precise alignment to a single fiducial nest.

These optimized designs significantly reduced the FSI head cost while improving installation ergonomics and system reliability. Fewer components also simplified the radiation qualification process for the final validation.

**Figure 10:**
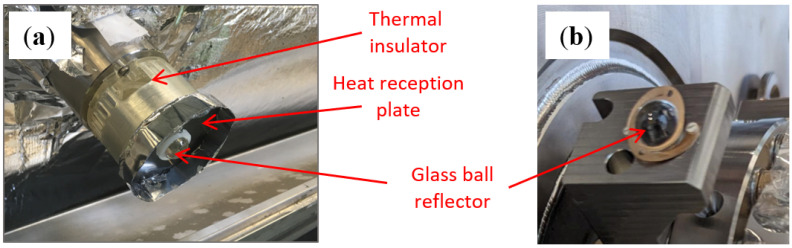
FT-FSI reflector support design for (**a**) the inner triplet and (**b**) the crab cavity.

The integration of glass ball retroreflectors was a crucial aspect in both applications. In the case of the crab cavity application, where the vacuum vessel is small and made of stainless steel, the reflector integration was straightforward. The glass balls were installed on stainless steel supports with conical fiducial nests and clamped using flexural springs (see Figure 10b).

Reflector integration in the inner triplet magnets was more complex. The ferritic steel cryostat is prone to rust, and its multi-layer thermal insulation traps water particles, risking cryo-condensation on glass reflectors. To prevent ice formation and signal loss, special supports were developed. These included a heat reception plate, which passively heated the reflectors using radiation from the cryostat, and a thermal insulator, which limited heat loss to the 2K cold mass (see Figure 10a) [43].

### 3.4. FSI Hydrostatic Leveling Sensor

**Figure 11:**
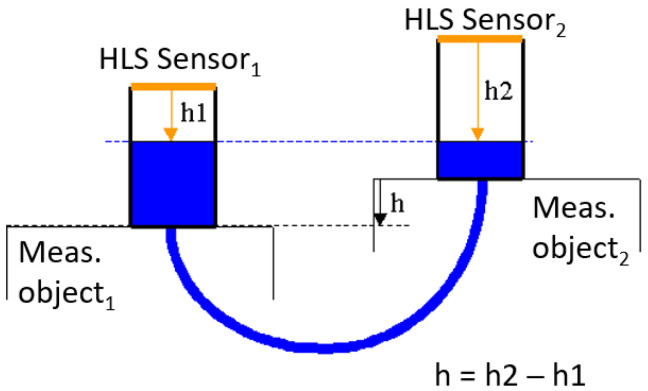
Hydrostatic levelling sensor network-operation principle: 2 HLS sensors connected to a single water network.

Micrometric leveling within FRAS is achieved by measuring multiple distances to the equipotential surface of water in a hydrostatic leveling network (HLN) using hydrostatic leveling sensors (HLS). According to the law of communicating vessels, the height difference between multiple HLS sensors, and thus the vertical alignment of objects, can be easily determined, as illustrated in Figure 11.

**Figure 12:**
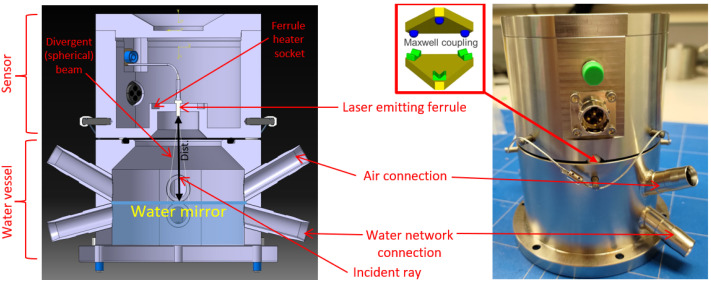
FT-FSI hydrostatic leveling sensor: (left) operation principle and (right) final prototype.

Figure 12 shows the FT-FSI HLS sensor developed at CERN. The system is constructed using a bare fiber ferrule for laser beam delivery, where incident rays perpendicular to the water, acting as a mirror, are reflected towards the fiber. This reflection provides the absolute ferrule-to-water distance, measured by the FT-FSI. The divergent Gaussian beam eliminates the need for precise vertical alignment of the sensor. Due to its spherical wavefront, the sensor can tolerate vertical alignment errors up to half of the laser’s beam divergence angle (in this case, using SMF28 single-mode fiber, with less than 8° to maintain sufficient optical power). However, vertical misalignment can introduce a cosine error in the HLS measurement, which is considered negligible, assuming a sensor alignment tolerance of ±1°.

Apart from its simple optical circuit, the HLS consists of several key sub-components essential to its operation. The HLN is a sealed system containing water and air, insulated from external environmental pressures, which could otherwise affect water levels and distort leveling results. The sensor is always paired with a “water vessel” connected to the HLN by two tubes. The bottom tube allows water flow, while the upper tube connects to the HLN air circuit. To maintain a closed circuit, the “water vessel” and sensor assembly are equipped with sealing gaskets.

The light-emitting ferrule is locally heated to prevent condensation on its tip, which could introduce measurement errors and attenuate the laser signal.

Precision in the HLS measurements is further ensured by its mounting system. The HLS is installed on the “water vessel” using a 3-ball Maxwell kinematic mount, providing installation repeatability within ±1 μm. This interface also serves as a local reference for HLS calibration, where the ferrule tip’s position (the origin of laser interference) is calibrated relative to the plane defined by the 3 balls. This design enables the interchangeability of sensors across “water vessels”, whose Maxwell interface sockets are fiducialized and aligned with the component’s reference frame.

The optical nature of FT-FSI HLS makes its design robust against electromagnetic noise and allows for long-distance connections to acquisition electronics, which are crucial for equipment operating in radiation-exposed environments, contrary to FOGALE nanotech [50] capacitive HLS sensors (see specification in [51], which are currently used in LHC inner triplet monitoring installations [13]).

### 3.5. FSI-Based Inclinometer

**Figure 13:**
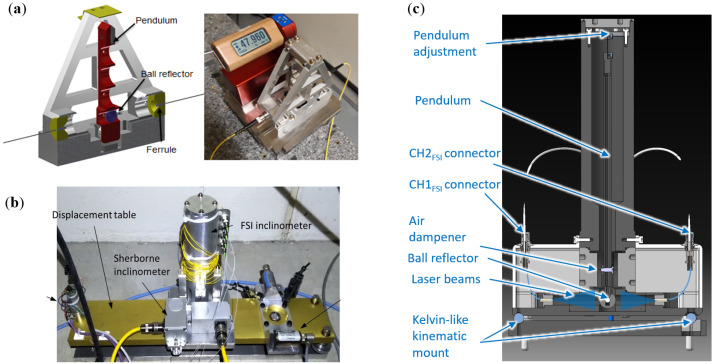
Three generations of FT-FSI-based inclinometer: (**a**) the first prototype of a single-axis inclinometer, equipped with a magnetic dampener; (**b**) the second prototype of a double-axis inclinometer, equipped with an oil dampener; (**c**) the third (final FRAS) prototype of an inclinometer with an air dampener. Adapted from [19].

An optical inclinometer consists of a pendulum suspended on a flexural hinge, with a single glass ball reflector placed at the bottom of the pendulum (see Figure 13a,c). The inclination angle is determined using double FT-FSI measurements of the distances between the ferrules and the glass reflector. The ferrules are located on both sides of the pendulum to differentially suppress thermal effects on the angle measurement. The objective for FRAS was to measure the angle with a precision of ±15 μrad.

The FT-FSI-based inclinometer underwent three prototype stages before reaching its final FRAS design. The first prototype (Figure 13a) tested whether the initial dimensions and magnetic damper could meet the precision requirement. The second (Figure 13b) featured a longer pendulum, bidirectional suspension with dual ferrule pairs for tip and tilt measurements, and tested a silicon oil damper as an alternative.

The final FRAS prototype (see Figure 13c) integrated lessons learned from previous versions. A longer pendulum was adopted after the initial design failed to achieve the required ±15 μrad precision. Single-axis operation was chosen because the bi-axial measurement approach introduced crosstalk between both axes, requiring complex calibration. Air-based dampeners were selected as they proved more suitable compared to magnetic and oil dampers, which were deemed problematic for their use and maintenance. To ensure durability, all sub-components were enclosed within a hermetic sensor body, protecting them from dust and humidity. An initial angle adjustment mechanism was incorporated to set the inclinometer’s “zero” angle during assembly and commissioning. For interchangeability, a two-ball Kelvin-like kinematic mount was implemented to ensure repeatable installation in the measured angle direction, while an additional vertical mechanism facilitated alignment in the perpendicular direction.

### 3.6. FSI Short- and Long-Distance Sensor

The short-distance FT-FSI sensors in the FRAS system are used for monitoring the longitudinal position of adjusted magnets, which are subject to longitudinal vacuum forces that can influence their position. These sensors are designed to be installed beneath magnets, fixed to the magnet support jack (which is secured to the ground), and measure longitudinal shifts of the magnet cryostat. The required measurement uncertainty of this sensor is 5 μm (k = 1).

**Figure 14:**
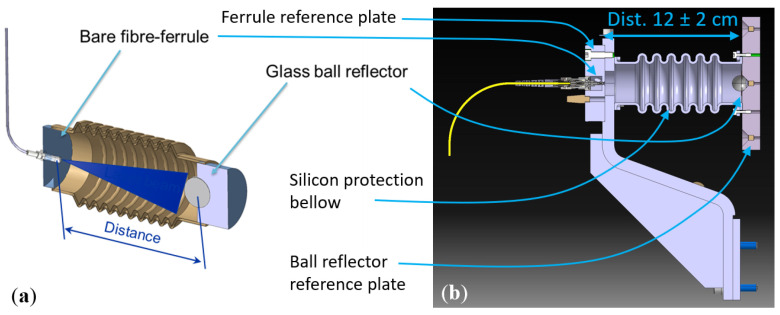
FT-FSI short-distance sensor: (**a**) operating principle and (**b**) FRAS sensor implementation.

The sensor’s operating principle and its FRAS implementation are illustrated in Figure 14. To simplify design and optimize costs, the sensor utilizes a divergent beam emitted by a bare fiber ferrule and a glass ball reflector. The final FRAS sensor implementation (see Figure 14b) includes several key elements in addition to optical components: the ferrule and reflector reference plates, which contain supplementary fiducials (additional SMR nests) for external referential measurements of ferrule and reflector positions using a laser tracker, and a silicon-made dust protection bellow, which maintains a clean optical volume, ensuring long-term, maintenance-free sensor operation.

**Figure 15:**
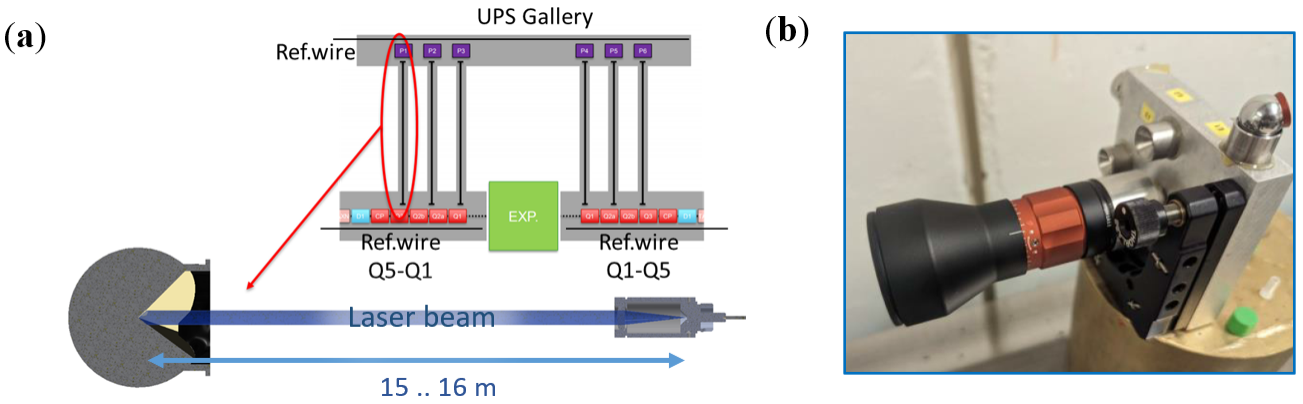
FT-FSI long-distance sensor: (**a**) operation principle including a collimated cylindrical laser beam with a retroreflector; (**b**) prototype with a wide (ϕ 20 mm) laser beam and optical measurement head.

The long-distance FT-FSI sensor is designed to transmit information about the radial position of FRAS components, determined using multiple stretched wires [16] across the experimental area. The long-distance sensor measurements link the left Q1–Q5 FRAS zone wire position to the right Q1–Q5 FRAS zone wire position through an adjoining “UPS gallery” wire (see Figure 15a). To meet FRAS alignment requirements (see Section 1), the measurement uncertainty of the sensor must be better than 20 μm (k = 1) over a distance of approximately 15 m.

## 4. Results

### 4.1. FT-FSI Measurement Systems: Test and Simulation Results

**Figure 16:**
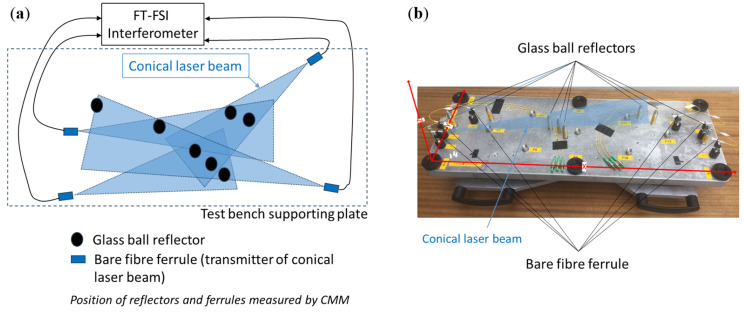
Absolute calibration test bench: (**a**) measurement principle; (**b**) picture of the bench (adapted from [18]).

Since the construction of the first FT-FSI interferometer prototype at CERN (see Section 2), the primary focus has been on the verification of the measurement accuracy of the interferometer unit. Due to the complexity of its sub-component processing algorithms, an analytical evaluation of the instrument’s measurement uncertainty proved to be challenging. Consequently, an empirical verification of the distance measurement uncertainty was conducted using a dedicated test bench [18] (see Figure 16).

The distance measurement verification involved comparative distance measurements between the FT-FSI system and those obtained using a CMM to measure the tips of the ferrules and glass reflectors. Based on these measurements, the combined standard uncertainty of the FT-FSI was estimated to be 4.9 μm for distances up to 0.7 m [18].

Additionally, the standard deviation of repeated measurements of a reflector, serving as an indicator of the repeatability of a single distance measurement, was found to be within ±1 μm for sharp, well-defined peaks in the FFT spectrum, and within ±2 μm for weaker peaks from more distant reflectors. These values were derived from 20 FSI measurements for every distance measured.

**Figure 17:**
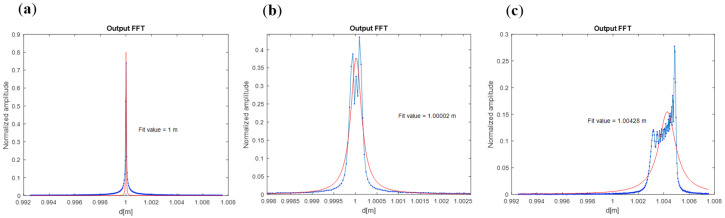
Example fits carried out on four measurements for a target placed at a distance of 1 m (blue is the FFT output and red is the Lorentzian fit function): (**a**) stable target with no vibrations; (**b**) low-amplitude vibrations (1 μm at 30 Hz); (**c**) target moving non-linearly outward of the collimator. Adapted from [21].

The measured distances are significantly influenced by vibration, the effect of which was therefore carefully analyzed. The aforementioned uncertainty estimation applies to a stable laboratory setup, where vibrations were considered negligible. However, introducing vibrations or movement can alter these values, as the FT-FSI results are highly sensitive to moving targets. Measurements and simulations conducted as part of a dedicated campaign [21] demonstrated that even small target displacements can be magnified by several orders of magnitude. The accuracy of the method depends not only on vibration amplitude but also on the target’s movement trajectory during the laser sweep, which affects the shape of the FSI output spectral response (see Figure 17 and [21]). The symmetry and shape of the spectral peaks significantly impact the quality of peak fitting, making FSI accuracy estimation more complex, as both the shift in the spectral peak and the fitting precision must be considered. The suitability of FT-FSI for applications that are subject to specific vibrations can be evaluated through the analysis of vibration spectra in conjunction with FSI system error simulations.

A subsequent measurement campaign, conducted in a vibration-free laboratory setup, focused on analyzing the main error sources in the FT-FSI measurements (see details in [20]). The main factor affecting accuracy was identified as the RMS fitting error on the gas cell peak retrieved from the gas cell photodetector signal, which is used to determine the laser sweep rate (α). Measurements using the first prototype and the portable test interferometer (see Section 2) showed that this fitting error alone results in a combined relative uncertainty in distance measurements of $3.1\times 10^{-6}$ m/m.

The fitting error of the gas cell peak signals is additionally influenced by distortions introduced during photodetection, signal amplification and analogue-to-digital conversion (ADC) within the FT-FSI setup. These distortions are systematic and can, therefore, be compensated, reducing the relative uncertainty in distance measurement to $3.06\times 10^{-7}$ m/m [20].

The key outcome from all the FT-FSI measurement campaigns at CERN was that the method is capable of achieving micrometric measurement uncertainty. However, the final uncertainty achieved is strongly dependent on the interferometer hardware, the chosen data processing methods, and the specific measurement application, particularly if vibrations are present. Additional studies based on field measurements are, therefore, necessary to estimate the uncertainty of specific FT-FSI configurations. For FRAS installations, vibration level analysis [21] indicates that, in the calm underground environment of CERN facilities, ground vibration amplitudes remain below 100 nm, which is negligible for FRAS sensor measurements. Furthermore, the likelihood of significant cultural noise sources in the LHC tunnel is considered negligible.

### 4.2. Tests of Multi-Distance and Multi-Sensor FT-FSI

**Figure 18:**
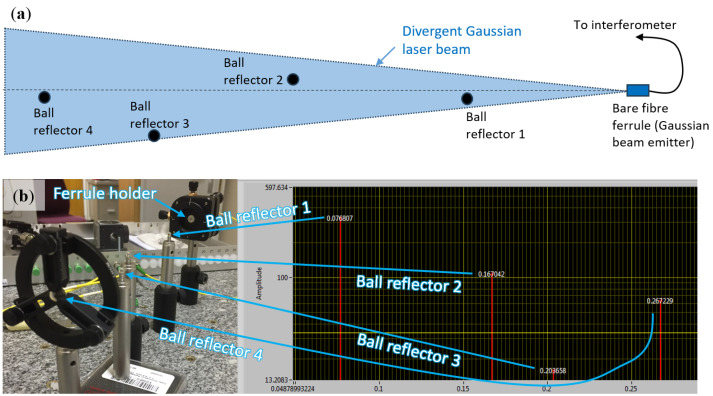
Multi-reflection tests with ball reflectors: (**a**) test bench layout and measurement principle; (**b**) photograph of the experimental setup and screenshot of the FT-FSI measurement result, highlighting the peaks corresponding to the reflectors.

**Figure 19:**
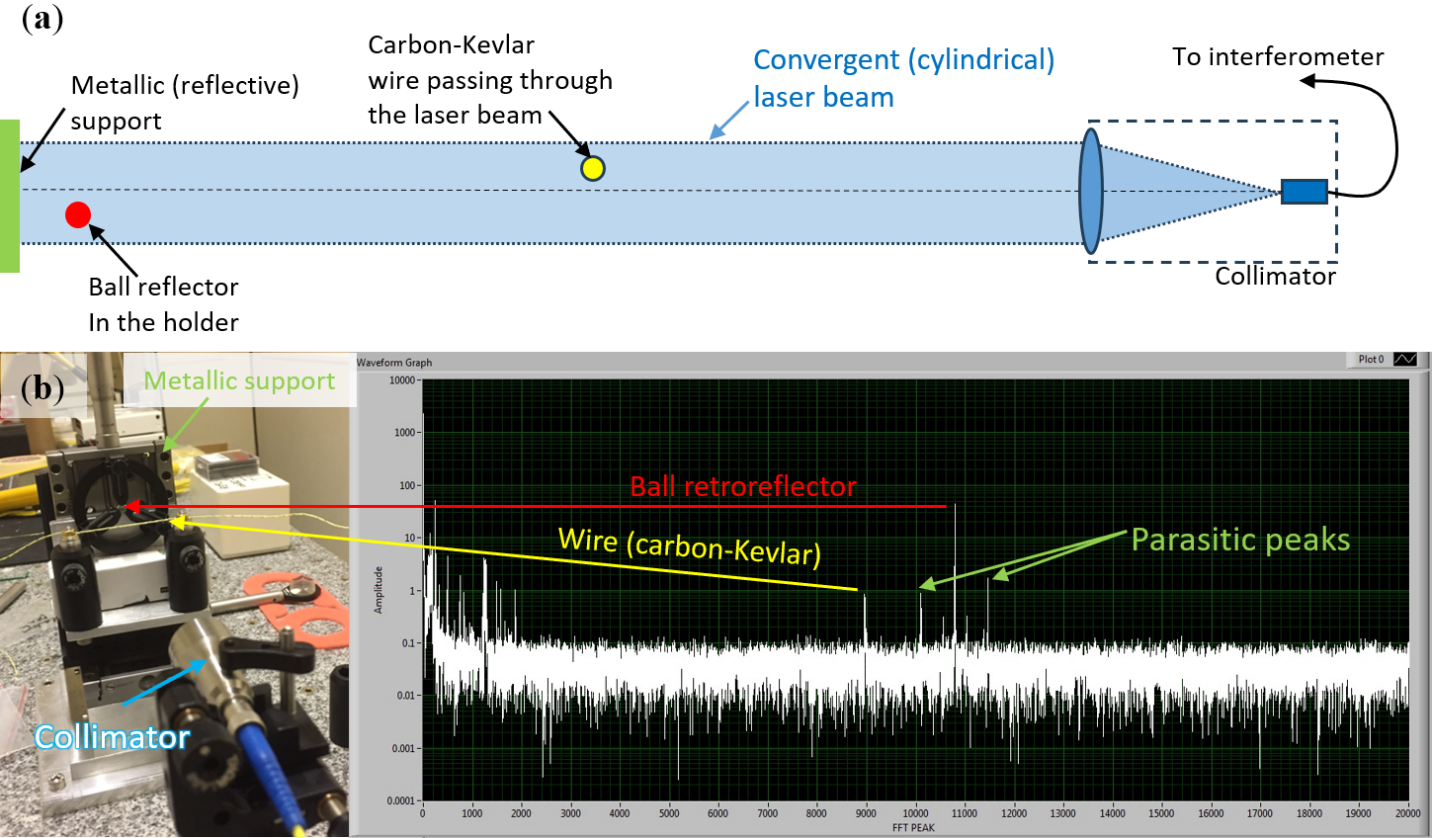
Multi-reflection tests on various objects: (**a**) test bench layout and measurement principle; (**b**) photograph of the experimental setup and screenshot of the FT-FSI measurement result, highlighting the peaks corresponding to various objects (glass ball, wire, and parasitic peaks from supports surfaces).

The ability to measure distances to multiple reflectors is one of the powerful capabilities of FT-FSI. Since the beginning of research and development on the FT-FSI measurement system at CERN, strategies for multi-reflector measurements were investigated in detail. The initial tests focused on the impact of the reflector type on such measurements. Figure 18 and Figure 19 show two test setups where the use of FT-FSI to measure the distance to glass ball reflectors, carbon-Kevlar wire, and surrounding metallic surfaces was compared.

The setup depicted in Figure 18 utilizes a ferrule tip enclosed within a dedicated tip–tilt adjustment holder to launch a divergent Gaussian laser beam toward four glass ball reflectors that are fully encompassed within the beam. Figure 18b (right) shows a screenshot from the FT-FSI data acquisition software used. The x-axis represents distance in meters (m), while the y-axis corresponds to FFT amplitude in arbitrary units. All four distances are measured simultaneously within a single laser sweep.

The closest reflector exhibits the highest amplitude, which is to be expected, as the laser beam power at the reflector’s position is greatest. The reflected signal amplitude decreases as the distance increases. Reflector number 3 has the lowest signal amplitude, as it is laterally displaced from the laser beam axis. In this off-axis position, the power of the emitted Gaussian beam arriving at the reflector is significantly lower than for on-axis reflectors 1, 2, and 4.

The setup shown in Figure 19 utilizes a convergent laser beam, emitted from a collimator, directed towards a stretched wire and a glass ball reflector. The collimator was employed to maintain a stable beam power throughout the volume of the emitted cylindrical beam, unlike a divergent conical beam, where the power decreases with the square of the distance. This increased the laser beam power density, enabling the detection of reflections from the wire, which is a weak reflector. Figure 19b (right) presents a screenshot from the FT-FSI data acquisition software, displaying the detected output FFT spectrum. The x-axis represents the FFT-bin number, while the y-axis corresponds to the FFT amplitude in arbitrary units. The peaks corresponding to the wire and glass ball reflector are clearly visible. The wire peak has a smaller amplitude, due to its lower reflectivity compared to the glass ball reflector. Since a cylindrical beam was used, providing a higher laser power density, parasitic reflection peaks from the aluminium surfaces of the side supports are also visible.

While increasing the FT-FSI laser power amplifies the reflected signals, enabling the detection of reflections from opaque or matte surfaces, such as from a wire (see Figure 19), it also raises the noise floor due to increased Rayleigh scattering in the fibers. It is essential to avoid placing any additional reflective surfaces near the measured reflector or surface to be measured, as this can introduce parasitic reflection peaks, as depicted in Figure 19b, that can compromise measurement accuracy.

**Figure 20:**
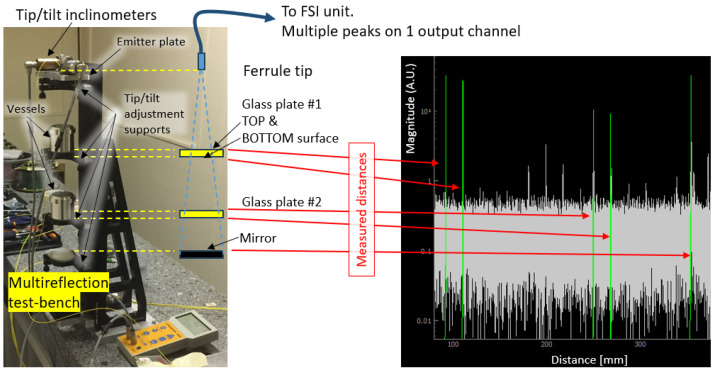
Multi-reflection test bench, where the distances from the fiber ferrule tip to the multiple surfaces were measured simultaneously.

To better characterize multi-reflection phenomena, the dedicated test bench depicted in Figure 20 was designed. The top “Emitter Plate” features a naked ferrule or light-emitting collimator, mounted on a tip–tilt support to allow for adjustments in the emission angle of the light. Beneath the “Emitter Plate”, two aluminium vessels are mounted on a tip–tilt adjustment jig, with each vessel fitted with a 12 mm-thick N-BK7 glass window, enabling light emitted from the “Emitter Plate” to pass through and reach the bottom silver-coated mirror plate. These vessels can be filled with different liquids to explore the reflection properties of liquid surfaces. The angles of all plates can be controlled using two inclinometers.

The measurements confirmed the FT-FSI’s capability for accurate measurements through multiple transparent media, with the light at an interface partly reflected (Fresnel reflections [25]). An example screenshot of interferometer distance reading can be seen on the right side of Figure 20. The first four peaks correspond to the top and bottom surfaces of the two transparent N-BK7 glass plates, while the last peak corresponds to the bottom mirror surface.

The thickness of the N-BK7 plates was measured using a screw gauge (measurement uncertainty of 1 μm, k = 2) and compared with values obtained via FT-FSI, using reflections from the top and bottom surfaces of the plates. To accurately determine the thickness from the optical path length (OPL), compensation was applied using the refractive index of the glass. For a 12 mm-thick plate, the corresponding OPL difference was approximately 18 mm.

It is important to note that the ordinary phase refractive index for monochromatic light in N-BK7 (e.g., *n* = 1.5007 at λ = 1550 nm [52]) is not suitable for OPL compensation in this context. This is because FT-FSI employs a laser that sweeps across the entire C-band (1530–1565 nm); thus, dispersion effects, such as the wavelength dependence of the refractive index [53], must be taken into account. Dispersion introduces a variation in time delay between signals from the reflecting target and the reference mirror in the interferometer (see Section 2, Equation (1)).

Instead of the phase refractive index, the group refractive index [54] was initially used. This index better reflects how fast a light pulse or wave packet propagates through a material when dispersion is present. For optical crystals and glasses, the group index in the visible and near-infrared ranges is typically slightly higher than the phase index. Applying a group refractive index of $n_{g}$ = 1.52 [52] to the FT-FSI measurements resulted in discrepancies of 8 μm for “Glass Plate #1” and 4 μm for “Glass Plate #2” when compared with screw gauge measurements.

The higher error observed for “Glass Plate #1” prompted a search for an “optimal” refractive index value tailored to the FT-FSI setup’s continuous frequency sweep across the optical C-band, rather than relying on the group index estimated for a wave packet. For this purpose, four N-BK7 plates of different thicknesses were measured using both OPL and screw gauge methods. The “optimal” refractive index was then calculated as the average of the ratio of OPL to mechanical thickness across all plates. This yielded a value of *n* = 1.5195, which is very close to the previously used group index. When applied to N-BK7 plate thickness measurements on the multi-reflection bench, the results fall well within the interferometer’s uncertainty.

Distance measurements to a water surface revealed that the reflections behaved in a similar way to those from a glass surface, with the main difference being the lower intensity of the reflected signal due to the lower refractive index of water ($n_{H_{2}O}$ = 1.3180 [55]) compared to the glass ($n_{N\_BK7}$ = 1.5007 [52]) at λ = 1550 nm) [25]. This result contributed to the design of the hydrostatic leveling sensor (see Section 3.4 and Section 4.3.2).

The bench also allowed the optimal inclination angle between the mirror surface and the ferrule axis for a ferrule with a Corning SMF28 fiber to be assessed. This fiber emits a conical Gaussian beam with a divergence angle of ±8° (see Section 3). At short distances of up to 10 cm and an emitted laser beam power of 1 mW, the distance peaks were easily distinguishable in the output spectrum of the CERN FT-FSI setup, achieving a signal-to-noise ratio (SNR) of approximately 15 to 20 dB, even with angular misalignments of up to ±8° to 10°. Even on the water surface, the distance peaks remained detectable in the FT-FSI output spectrum with angular misalignments of up to ±6° to 7°.

The feasibility of FT-FSI measurements through a water layer could also be investigated using this test bench. Laser light in the infrared C-band (1530–1565 nm) is highly attenuated by water (absorption coefficient of $10^{3}$ to $10^{4}\;m^{-1}$ at λ = 1550 nm [56]). Therefore, the goal was to establish the maximum thickness of water through which reflections could still be measured. At a nominal emitted power of 1 mW, a 4 mm thick water layer significantly attenuated the signal reflected from the bottom glass surface of the water test vessel. Increasing the emitted power led to a linear increase in the depth of water through which measurements could still be made.

**Figure 21:**
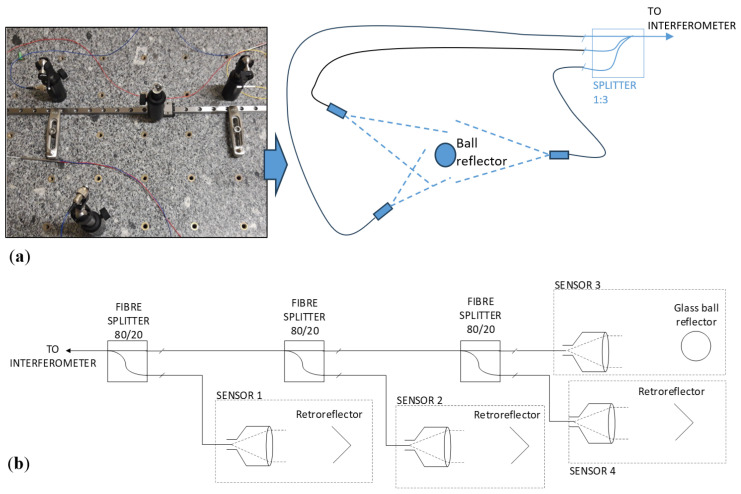
Multi-sensor configuration using FT-FSI: (**a**) an example of the triangulation measurement of a glass reflector using three bare fiber ferrules (left) and its connection schematic (right); (**b**) the possible connection of 4 distance sensors to a single interferometer channel. Adapted from [18].

The FT-FSI’s ability to measure multiple distances simultaneously enables the connection of multiple sensors to a single interferometer channel (see Figure 21). By utilizing fiber splitters, it is possible to simultaneously measure different distances from various sensing devices. Tests of these sensor configurations demonstrated the proper operation of FT-FSI under these conditions. Figure 21a illustrates an example of triangulation measurement of a glass reflector using three bare fiber ferrules.

It is important to note that such a configuration is effective only when the distances from each target are distinguishable, i.e., when the FFT peaks associated with various distances do not overlap. Attention must also be paid to the lengths of the connecting fibers and the return loss of the connectors [57]. Each connector can introduce back reflections that interfere with the other reflected signals, appearing in the FT-FSI output spectrum as additional beat frequencies. Consequently, selecting fiber segment lengths similar to the distances to be measured may result in overlapping FFT peaks. To minimize these risks, it is best to use connectors with low return losses (<−40 dB), such as angled physical contact (APC) or ultra-physical contact (UPC) connectors [57].

**Figure 22:**
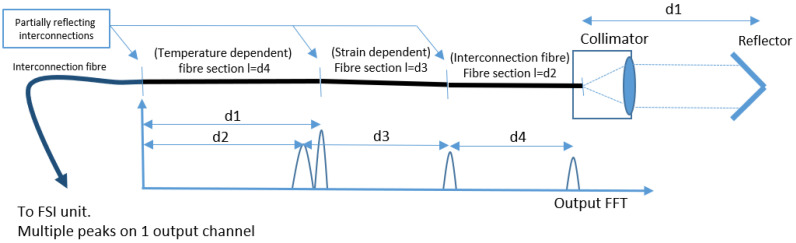
Conceptual schematic of the possibility to measure multiple distances within serially connected fiber sections using a single FT-FSI channel.

Back reflections from fiber interconnections can be advantageous when establishing a distributed in-fiber measurement system using FT-FSI. Such a system allows for the measurement of various physical quantities by detecting changes in the lengths of fiber sections. Figure 22 illustrates a conceptual layout where multiple fiber sections measuring various physical properties are separated by partially reflective interconnections within a single fiber, all measured using a single FT-FSI channel.

Each section consists of semi-reflective entry and exit mirrors to create interference beat frequencies relative to their length. With the micrometric precision of FT-FSI, these measurements can be applied to various fiber-based sensor applications, such as deformation and strain monitoring [58], temperature measurements, and load sensing. Integrating a network of such sensors within a single fiber also reduces the cost of such a measurement system.

### 4.3. Robust FT-FSI Sensors for Use in Harsh Environments: Test Results

#### 4.3.1. FRAS Internal Monitoring Sensors

The operation principle of the FSI monitoring within cryostats at CERN is based on direct measurement between the tip of a fiber ferrule and a glass ball reflector. A modified collimator configuration with an aspheric lens can be installed after the ferrule to modify the laser beam divergence angle (see Section 3.3).

**Figure 23:**
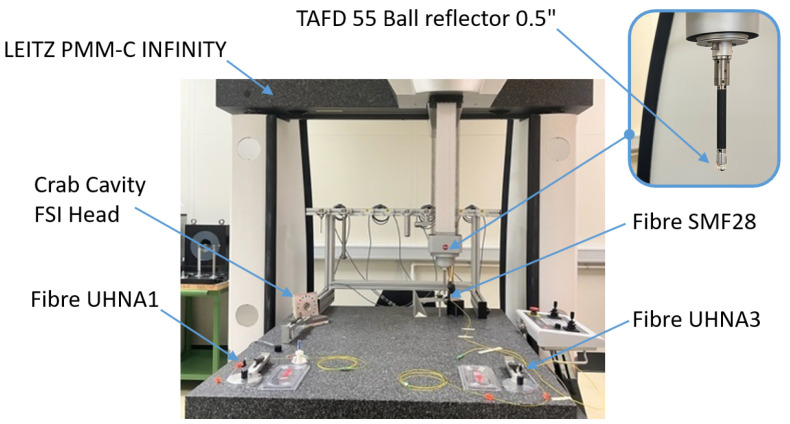
CMM measurements of various laser beam emitters using a 0.5″ glass ball reflector [59].

In addition to the accuracy assessment during comparative measurements of CMM and FT-FSI on the test bench (see Section 4.1), a detailed characterization of the FSI heads was carried out to assess their measurement accuracy across the entire footprint of divergent beams. A series of experiments was conducted using a dedicated CMM-based setup (see Figure 23), where the position of a ball reflector within the laser beam illumination field was compared with parallel FT-FSI measurements [59]. The setup included a TAFD 55, 0.5″ ball reflector attached to the CMM measurement head and four types of laser emitters: an SMF 28 fiber ferrule (representing the inner triplet FSI head), the crab cavity FSI head, and two ferrules with ultra-high numerical aperture (UHNA) fibers [60]. The UHNA fibers were investigated for their wide-angle conical beams (the theoretical divergence angles of the UHNA1 and UHNA3 fibers used were 32.5° and 41°, respectively). The CMM used in this campaign was a Leitz PMM-C Infinity CMM, with a maximum permissible error ($MPE_{E}$) of 0.3 + L/1000 μm [61].

Comparative measurements for the SMF28 fiber were taken at distances of 200, 400, 600, and 1100 mm within the laser beam field of view. For the crab cavity head, measurements were conducted at the nominal operating distance of 700 mm. The mapping of measurements using the SMF28 fiber was carried out because of the extensive use of such fibers in various CERN measurement systems (inclinometers, long- and short-distance sensors, calibration equipment, etc.).

**Figure 24:**
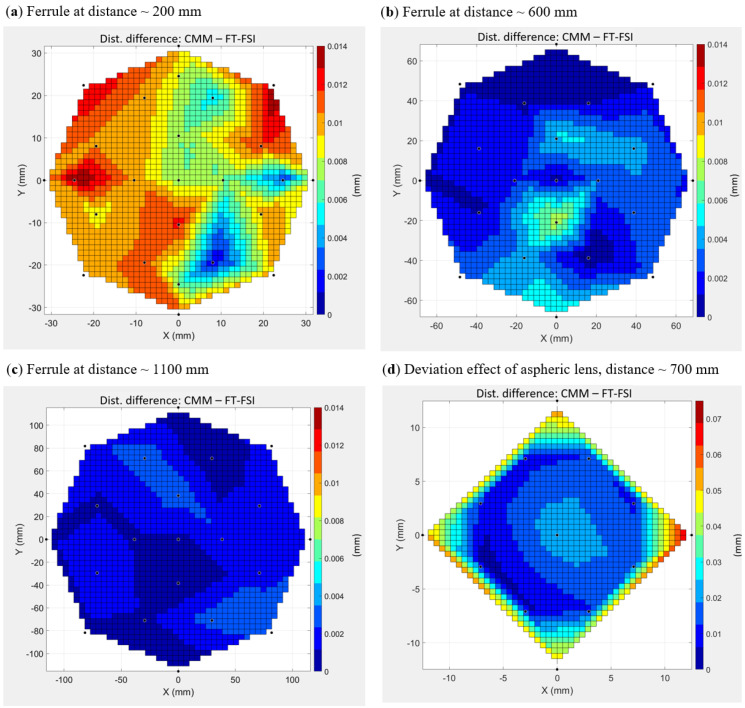
CMM to FT-FSI comparative measurements for inner triplet and crab cavity FSI heads [59]: (**a**) SMF28 measurements at 200 mm (measurements with higher variance due to parasitic reflections); (**b**) SMF28 measurements at 600 mm; (**c**) SMF28 measurements at 1100 mm; (**d**) the measurement of the crab cavity FSI head at 700 mm (compensated for the lens refractive index but with OPL aberrations due to the enlargement of the spherical wave radius being uncorrected).

Figure 24a–c shows the measurement results relevant to the inner triplet FSI head, comparing the measured distance differences between the CMM and SMF28 fiber FT-FSI measurements, while Figure 24d shows the results of the crab cavity head measurements. The X–Y position in these figures indicates the lateral offset of the reflector from the pointing axis of the laser beam during the measurement, with the plotted values being the absolute difference in mm measured between the CMM and FT-FSI. The measurement conclusions were as follows:

- Most FSI measurements, when compared with CMM data, confirm the previously estimated FT-FSI uncertainty of approximately 4.5 μm within the 0.1 m to 1.1 m range [59].
- The first measurement with the SMF 28 fiber (depicted in Figure 24a) shows a larger variation in CMM to FT-FSI difference. This was due to excessively high FT-FSI power, causing significant parasitic reflections from shiny surfaces (reflector nest and CMM handling bar) that affected the identification of FFT peaks. Reducing the FT-FSI signal power mitigated this effect and lowered the CMM-FT-FSI difference dispersion to 4 μm [59]. This confirms the importance of designing sensor equipment to minimize parasitic reflections.
- At some points in the measured aperture, jumps in the CMM to FT-FSI difference were observed (e.g., 9 μm in Figure 24b), that can again be explained by parasitic reflections.
- In conditions where parasitic reflections are minimized, the reflector’s position within the laser beam field of view did not impact the measurement accuracy for bare SMF28 fiber measurements.
- For the crab cavity FSI head measurements (cf. Figure 24d), where an aspheric lens was used (see the design details in Figure 9b), the reduced divergence angle of the laser beam caused an enlargement of the spherical wave radius emitted from the head. This effect was visible as a gradual increase in the CMM to FT-FSI difference with increasing distance from the aperture center. This known effect can be corrected through FSI head calibration.

**Figure 25:**
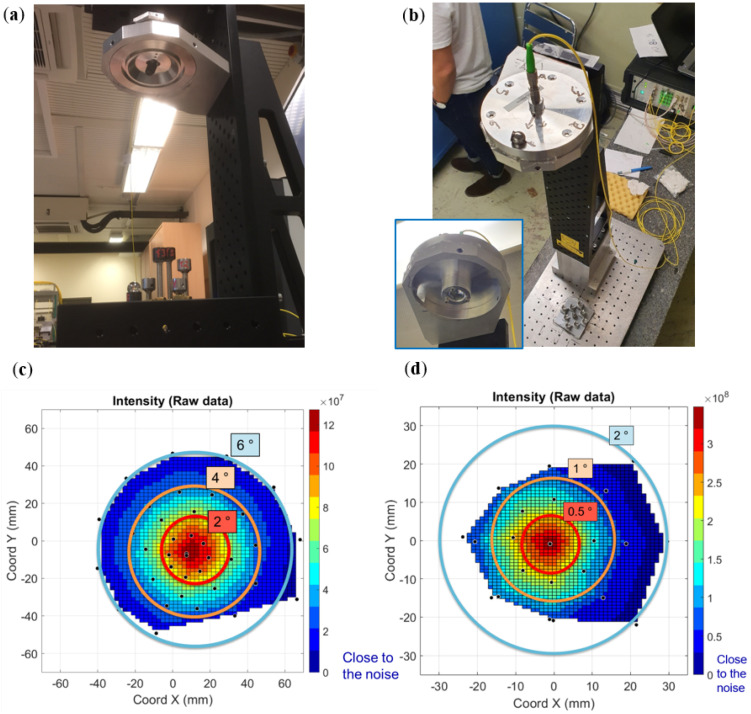
(**a**) Inner triplet FSI head calibration bench; (**b**) crab cavity FSI head calibration bench; (**c**) inner triplet FSI head field of view and beam divergence measurement results; (**d**) crab cavity FSI head field of view and beam divergence measurement results.

To prepare the FSI heads for future measurements on real objects, the FSI measurement origin (the position of the ferrule tip in the external metrological frame of the FSI head) and any effect of the aspheric lens-induced OPL change needed to be calibrated. Such a calibration was performed using special set-ups (see Figure 25a,b), allowing parallel measurements with a laser tracker and FT-FSI. The coordinates of the fiducial nests of the FSI head (used as external references) and those of the fiducial nests of the reflectors were first measured using the laser tracker. The FT-FSI distances to the glass ball reflectors located in the same fiducial nests were then measured. By combining these data, the coordinates of the FT-FSI origin relative to the external fiducial nests were determined.

During the FSI head calibration, the laser beam opening angle, which defines the field of view of both heads, was verified to ensure that measurements would be possible at all required positions of the reflectors (see Section 3.3). A divergent beam cone angle of 12° was required for the inner triplet FSI head, and 2.2° was required for the crab cavity FSI head. Figure 25c,d shows the results of these measurements, where the field of view (and divergent beam cone angle) is indicated on the reflection intensity graphs. The main conclusions from these measurements are as follows:

- The intensity distribution within the measurement cone exhibits a Gaussian-like profile. The farther from the cone’s center, the lower the return intensity (see Figure 25c,d for details).
- A divergent beam opening angle of approximately 12° (2× 6°) was confirmed for the inner triplet FSI head.
- A beam opening angle of approximately 2.2° (2× 1.1°) was confirmed for the crab cavity FSI head.
- Increasing the nominal 1 mW-emitted laser power can enlarge the field of view for the heads.

In parallel with the validations performed in laboratory conditions, prototypes and final design pre-series of the FSI heads were used for internal monitoring measurement tests on the inner triplet and crab cavities. These tests validated the measurements under vacuum and cryogenic conditions. Further details on these test campaigns can be found in [48,62].

#### 4.3.2. FSI Hydrostatic Leveling Sensor

The FSI-HLS sensor for FRAS underwent extensive testing between 2018 and 2024, as it is a critical component for maintaining the precise horizontal leveling of accelerator components over a 420 m distance. To ensure its reliable future operation, four main testing phases were conducted, as described below.

**Phase 1** focused on testing HLS sensor prototypes under laboratory conditions, assessing the impact of environmental vibrations, extreme angular misalignments of the sensor ferrule relative to the water level, water contamination (dissolved corrosion residues and algae), and water evaporation effects. This phase also looked at the long-term laboratory comparison between capacitive and FSI-HLS sensors.

The tests showed no significant impact of algae or rust contamination in the water on FSI-HLS measurements, as algae settled at the bottom of the water vessels and the rust did not form attenuating layers. Water evaporation, however, caused a gradual measurement drift due to the changing water levels, emphasizing the need for a sealed HLS network, as in open vessels, the distance changes due to evaporation were in the range of 12–17 nm/s.

The maximum allowed misalignment error for FRAS sensors was specified to be ±1°. Deviations within ±5° were found to still provide acceptable signals, confirming compatibility with the FRAS specifications.

Surface vibrations caused by striking the vessel led to large measurement errors or signal loss [21]. In the absence of forced vibrations, laboratory tests showed standard deviations of 2 μm during night-time, i.e., when human sources of noise in the vicinity were minimal, and 3.3 μm during daytime. These values fall well within the acceptable range for future FRAS FSI-HLS applications. Analysis also revealed a Gaussian noise distribution, allowing signal averaging to be used to improve measurement accuracy in the presence of vibrations.

The comparative study between FSI-HLS and capacitive sensors was conducted over 300 h, with significant water level changes introduced every 50 h. The FSI-HLS measurements matched those of the capacitive sensors, with a maximum deviation of just 3 μm. These initial results confirmed the accuracy and demonstrated the correct measurement performance of FSI-HLS sensors for FRAS.

**Phase 2** concentrated on comparative measurements between capacitive HLS and FSI-HLS sensors on a 110 m LHC-representative HLS network and an assessment of the impact of vibrations in a low-noise environment. Two pairs of capacitive and FSI-HLS sensors were installed: one at the end and one at the midpoint of the 110 m water network tube. The network was located in an underground tunnel at CERN, providing a realistic noise and temperature environment for the testing of micrometric measurement systems foreseen for future accelerator installation.

**Figure 26:**
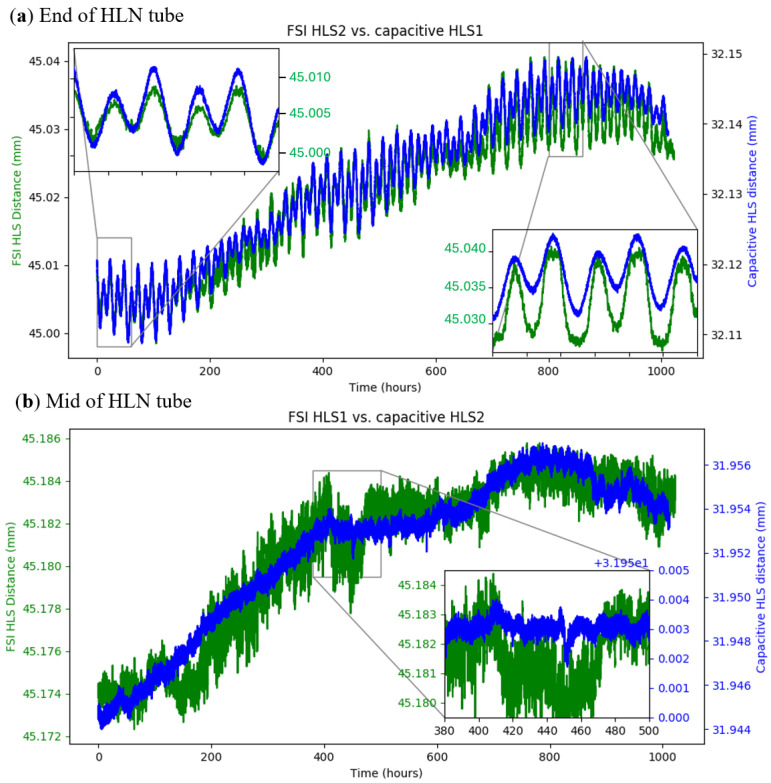
Long-term comparative measurements of capacitive and FSI-based HLS sensors connected to a 110 m hydrostatic leveling network: (**a**) sensor data from the HLN tube end, with the tidal effects of the moon on the water in the network clearly visible; (**b**) sensor data from the middle of the HLN tube.

Figure 26 shows the results of Phase 2 measurements. In agreement with the observation in Phase 1 in the laboratory, the curves obtained with FSI-HLS sensors closely matched those of their capacitive counterparts, with a difference of only 3 to 4 μm. An interesting observation was the effect of the moon’s tidal forces on the HLS water network, which were seen as oscillations in the measurements from sensors at the end of the tube (cf. Figure 26a). As expected, this tidal effect was not visible at the midpoint of the HLS network since the moon’s gravitational influence tilts the water along the length of the tube but has minimal impact at its centre. This confirms the sub-micrometric resolution of FSI-HLS sensors.

As expected, the noise in the FSI-HLS measurement signal (cf. Figure 26b) in the more stable environment of the underground tunnel was found to be significantly lower than in the laboratory tests conducted during Phase 1, with a standard deviation of only around 200 nm.

**Figure 27:**
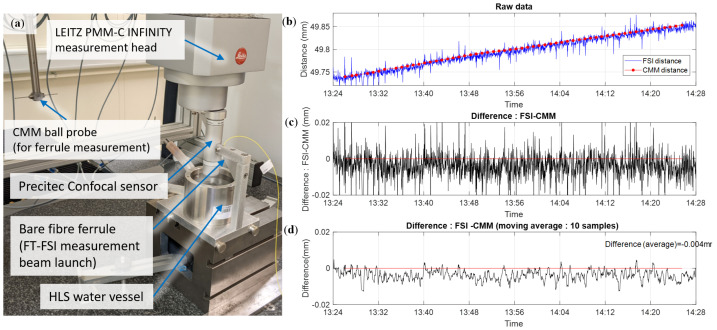
FSI-CMM confocal sensor tests: (**a**) view of the test setup; (**b**) optical CMM measurement comparison; (**c**) FSI-CMM difference; (**d**) FSI-CMM difference (10-sample moving average).

**Phase 3** focused on validating the accuracy of the water surface measurement through comparative tests with a CMM machine. To perform this validation, the test setup depicted in Figure 27a was constructed. It consisted of a Leitz PMM-C Infinity CMM ($MPE_{E}$ = 0.3 + L[mm]/1000 μm [61]) equipped with a Precitec LR confocal sensor [63]. The maximum offset between the PMM-C tactile probe and the Precitec sensor was declared to be less than 1 μm [64], leading to a conservative $MPE_{E}$ estimate for the full setup (PMM-C Infinity + Precitec LR) of 1.3 + L[mm]/1000 μm.

The setup allowed the parallel calibration of the tip of the FT-FSI ferrule (where the FT-FSI measurement origin was determined through CMM ball probe surface scanning) and the Precitec sensor (where the high-grade reference ball was first scanned by the optical sensor and then by the CMM ball probe to determine the measurement origin of the sensor). These combined measurements linked the coordinate systems of the Precitec probe and the FT-FSI ferrule, allowing for comparative measurements of the water distance using both techniques.

Figure 27 shows the measurement results, where the difference in raw data between FSI and CMM is visible in Figure 27c, and the same data filtered with a moving average of 10 samples are shown in Figure 27d. Throughout the measurement period, the average difference between the CMM-Precitec and FT-FSI was less than 4 μm, which fell within 4.92 μm, derived as the combined standard uncertainty of FT-FSI (4.9 μm, see Section 4.1), and CMM-Precitec ((1.3 + 50/1000)/3 = 0.45 μm ($MPE_{E}$ was considered equivalent to a 3σ measurement).

**Phase 4** of FSI-HLS testing was conducted on the single component test (SCT) mock-up at CERN [65], where the dynamic behavior of all FRAS sensors and control software was evaluated. Step response scenarios, including motions from 0.04 mm to 1 mm (the latter exceeding FRAS requirements) with a 9-s acceleration period, were tested. For a 1 mm step, an overshoot of 50 μm was observed. This was expected due to the Doppler effect on FSI measurements [21] and the time required for water level stabilization in the HLS network. This overshoot dissipated within 10–15 s, which was similar to that observed in current LHC inner triplet monitoring [13] installations. These tests confirmed the absence of abnormal behavior in FSI-HLS sensors under dynamic conditions.

#### 4.3.3. FSI-Based Inclinometer

**Figure 28:**
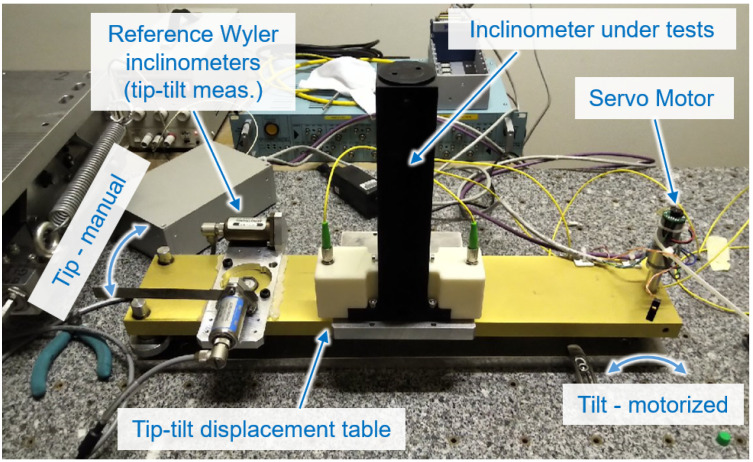
Inclinometer test bench: tip–tilt adjustment table equipped with a motor (for tilt adjustment), allowing for the cyclic change in the tested inclinometer tilt, and comparison with a reference inclinometer.

The primary test for the three inclinometer generations investigated (see Section 3.5, Figure 13) was a comparative cycling test on an inclinometer test bench (depicted in Figure 28). This allowed the cyclic tilting of the tested inclinometer and comparison with a reference Wyler ZEROTRONIC-3 inclinometer [66]. The reference interferometer provided a measurement precision of 5 μrad. The sensors were continuously tilted forward and backward by 6 mrad to 7 mrad over several days.

The third prototype (cf. Figure 13c) met the precision requirement for FRAS, and its damper construction allowed for easy handling, installation, and maintenance. To better assess the measurement error of this final prototype, the reference inclinometers were replaced with Sherborne LSOC-1 units [67] (range of ±1° and measurement precision of 0.5 μrad), and the measurements were performed at the underground facility. The standard deviation of the error between the FSI inclinometer and the reference readings for 35 days was approximately 5 μrad [19].

The final part of inclinometer testing was conducted on the SCT mock-up [65], where the integration and installation procedures of the sensor, as well as the inclinometer’s operation under dynamic conditions, were validated to meet the FRAS requirements.

#### 4.3.4. FSI Short- and Long-Distance Sensor

Short-distance sensors (see Section 3.6, Figure 14), used for the longitudinal position monitoring of FRAS components, measure the distance between a fiber ferrule and a glass reflector. Since the properties of this measurement configuration were validated during test bench measurements (Section 4.1) and FSI internal monitoring head tests (Section 4.3.1), no additional laboratory validations were required. The only tests conducted were on the SCT mock-up [65], validating the integration and installation procedures of the sensor and confirming that it met the FRAS operational requirements.

As introduced in Section 3.6, long-distance sensors were used to transfer information onto the radial alignment of components between the left and right zones of FRAS, on either side of an LHC experiment, through the “UPS Gallery”. To meet FRAS radial alignment requirements (see Section 1), the accuracy of the long-distance sensor must be better than 20 μm for measurements over a 15 m distance. Based on the analysis of error sources in FT-FSI measurements from the interferometer setup [20], as recalled in Section 4.1, the interferometer (in stable measurement conditions) was characterized by an uncertainty of $3.1\times 10^{-6}$ m/m for distance measurements. While full validation of the FSI long-distance sensor was not completed at the time of preparing this manuscript, this value, when applied to a 15 m distance, leads to a measurement uncertainty of 46.5 μm, which does not fulfil the sensor requirement of better than 20 μm.

This uncertainty could be reduced by compensating for systematic effects of the interferometric setup, such as fitting errors in the gas cell peak signals and non-linearities in the front-end circuitry (see Section 4.1 and [20]), potentially lowering the relative combined uncertainty in the distance measurement to $3.06\times 10^{-7}$ m/m, which corresponds to an absolute uncertainty of approximately 4.6 μm for a 15 m measurement.

**Figure 29:**
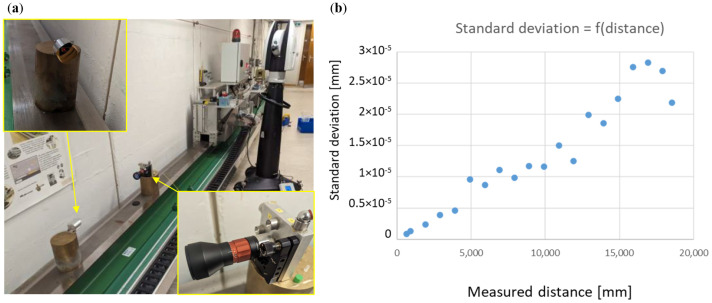
FT-FSI long-distance sensor tests: (**a**) prototype optical measurement line; (**b**) test results as a standard deviation of the function of the measured distance.

To verify these estimates and validate the prototype design of the sensor optics, laboratory tests were conducted with a long-distance sensor prototype (see Figure 29a). A series of comparative measurements between FT-FSI and a laser tracker was performed over 20 distances, ranging from 0.6 to 18.5 m.

For each of the 20 distances, 21 FT-FSI measurements were taken to analyze the behavior of measurement noise. Figure 15b shows the standard deviation calculated from these measurements, which serves as an indicator of the noise level and measurement precision. As the distance increased, the standard deviation of the noise in the measurement rose to a value of 23 μm at 15 m. This behavior was expected as fluctuations in the air’s refractive index (caused by air flow and convection in the measurement facility) increasingly affect the measured OPL with distance. Additionally, the fitting error of the gas cell impacted the measurement accuracy proportionally to the increase in the measured distance.

While these results are promising, a measurement uncertainty below 20 μm over a 15 m distance has not yet been demonstrated. To further improve precision, the number of sensors between the LHC tunnel and the ‘UPS Galleries’ (see Section 3.6, Figure 15) will be doubled from three to six on each side of the FRAS, thereby increasing both the number of sensors and observations. Additionally, increasing the number of measurements over time (given the quasi-static nature of the system) is expected to further improve uncertainty and support achieving the required accuracy.

## 5. Summary

The central challenge in delivering the CERN fully remote alignment system was *how to develop an absolute-distance-based micrometric measurement system for 3D alignment monitoring that can achieve resolutions of 1 μm and an accuracy below 5 μm for hundreds of measured objects in a harsh environment?*

The selection of FT-FSI for this task was based on five main factors: its capability for absolute measurements, its insensitivity to light-intensity variations, its resistance to environmental disturbances, e.g., electromagnetic noise, its lower cost through the use of optical fibers instead of copper cables, and the need for periodic calibration.

Its scalability enabled the streamlining of the acquisition system, significantly reducing infrastructure costs. The initial interferometer prototype, measuring up to eight channels, had a cost of approximately CHF 5000 per channel, which is comparable to commercial interferometers. However, the final 256-channel system reduced this cost by well over a factor of ten to only some CHF 500 per channel. Further optimization is still possible, particularly if the sweeping laser system can be simplified, as this remains a major cost driver.

Ensuring the long-term robustness of sensors over a 15-year operational period is critical. Therefore, the FRAS FSI sensors were designed with simplicity in mind, featuring no moving parts, a minimal number of components, and built using radiation-hard materials. The test results presented in this work demonstrate that the proposed solutions meet the stringent operational requirements of the FRAS system. Such a system could be readily adapted for other large-scale or dimensional metrology applications.

While the FT-FSI accuracy has already been demonstrated to satisfy most FRAS requirements, it could be further improved by compensating for system non-linearities. Although not critical for the FRAS, further research on this is planned for future applications, such as monitoring mechanical deformation in the next generation of particle accelerators. The main limitation of using a single sweeping laser for FT-FSI measurements is its sensitivity to vibrations and reflector motion. Future systems, where this is more of an issue than for the FRAS, may therefore benefit from dual-laser configurations, frequency mirroring, or cost-effective modulation techniques.

In 2025, FT-FSI and FSI-based sensors will undergo their final validation at CERN’s inner triplet string test facility. Here, one full magnetic assembly consisting of six magnets and ancillary components, in a similar configuration to their final home in the LHC tunnel, will be tested in a surface hall. This will provide a final opportunity to optimize FRAS installation procedures and verify the performance of all FRAS components ahead of full FRAS deployment in the LHC tunnel in 2027.

## Data Availability

The data supporting the findings of this study are available from the corresponding author upon reasonable request.

## Abbreviations

The following abbreviations are used in this manuscript:

ADC	Analog-to-Digital Converter
APC	Angled Physical Contact (Fiber Connector Type)
CMM	Coordinate Measuring Machine
DIOT	Distributed I/O Tier
ECDL	External Cavity Diode Laser
EMC	Electromagnetic Compatibility
FCC	Future Circular Collider
FEC	Front-End Computer
FESA	Front-End System Architecture
FFT	Fast Fourier Transform
FRAS	Full Remote Alignment System
FSI	Frequency Scanning (or Sweeping) Interferometry
FT-FSI	Fourier Transform-based Frequency Sweeping Interferometry
GPU	Graphics Processing Unit
HL-LHC	High-Luminosity Large Hadron Collider
HLN	Hydrostatic Leveling Network
HLS	Hydrostatic Leveling Sensor
LHC	Large Hadron Collider
MS/s	Mega Samples per Second
NA	Numerical Aperture
PC	Physical Contact (Fiber Connector Type)
PCB	Printed Circuit Board
SMF	Single-Mode Fiber
SNR	Signal-to-Noise Ratio
SMR	Sphere Mounter Reflector
TID	Total Ionizing Dose
UHNA	Ultra-High Numerical Aperture
UPC	Ultra Physical Contact (Fiber Connector Type)
WPS	Wire Position Sensor
